# Supplementary material for: Timing of Smarcb1 and Nf2 inactivation determines schwannoma versus rhabdoid tumor development
Source: Nat Commun. 2017 Aug 21;8:300. doi: 10.1038/s41467-017-00346-5 (PMC5563506; doi:10.1038/s41467-017-00346-5)
Supplement: Supplementary file 1 — Supplementary Information [file 41467_2017_346_MOESM1_ESM.pdf]

Description of Supplementary Files

File Name: Supplementary Information
Description: Supplementary Figures and Supplementary Table

File Name: Peer Review File

a

	P0-CreB;Smarcb1$^{flox/flox}$	*P0-CreB;Smarcb1*$^{flox/+}$	*Smarcb1*$^{flox/flox}$	*Smarcb1*$^{flox/+}$
**Alive at weaning**	1.4%	30.2%	30.1%	38.3%
**Pups found dead at birth**	59.6%	19.1%	11.3%	9.9%
**Expected**	25%	25%	25%	25%

b

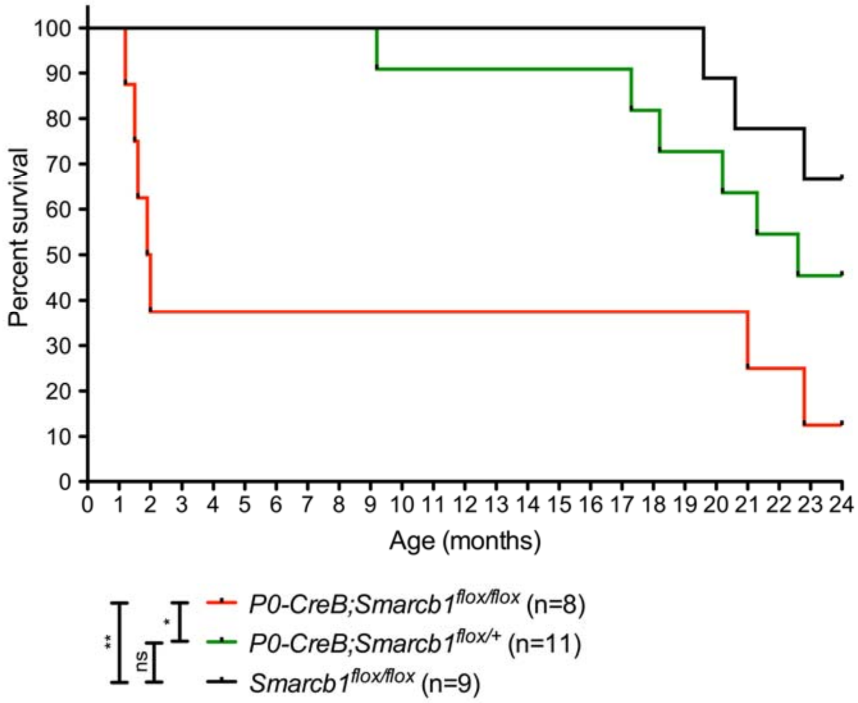

Supplementary Figure 1. *P0-CreB;Smarcb1*$^{flox/flox}$ mice present a low viability and a survival median of 2 months of age. (**a**) Genotype distribution of mice at 3 weeks of age (n=569) or from pups found dead at birth (n=141) born from crossings of *P0-CreB;Smarcb1*$^{flox/+}$ and *Smarcb1*$^{flox/flox}$ mice. (**b**) Kaplan-Meier curve representing the percent survival of *P0-CreB;Smarcb1*$^{flox/flox}$ (n=8, median survival = 2 months), *P0-CreB;Smarcb1*$^{flox/+}$ (n=11, median survival = 22.6 months) and *Smarcb1*$^{flox/flox}$ (n=9, median survival >24 months) mice versus age in months. ns (non significant), * ($p<0.05$), ** ($p<0.01$); log-rank (Mantel-Cox) test.

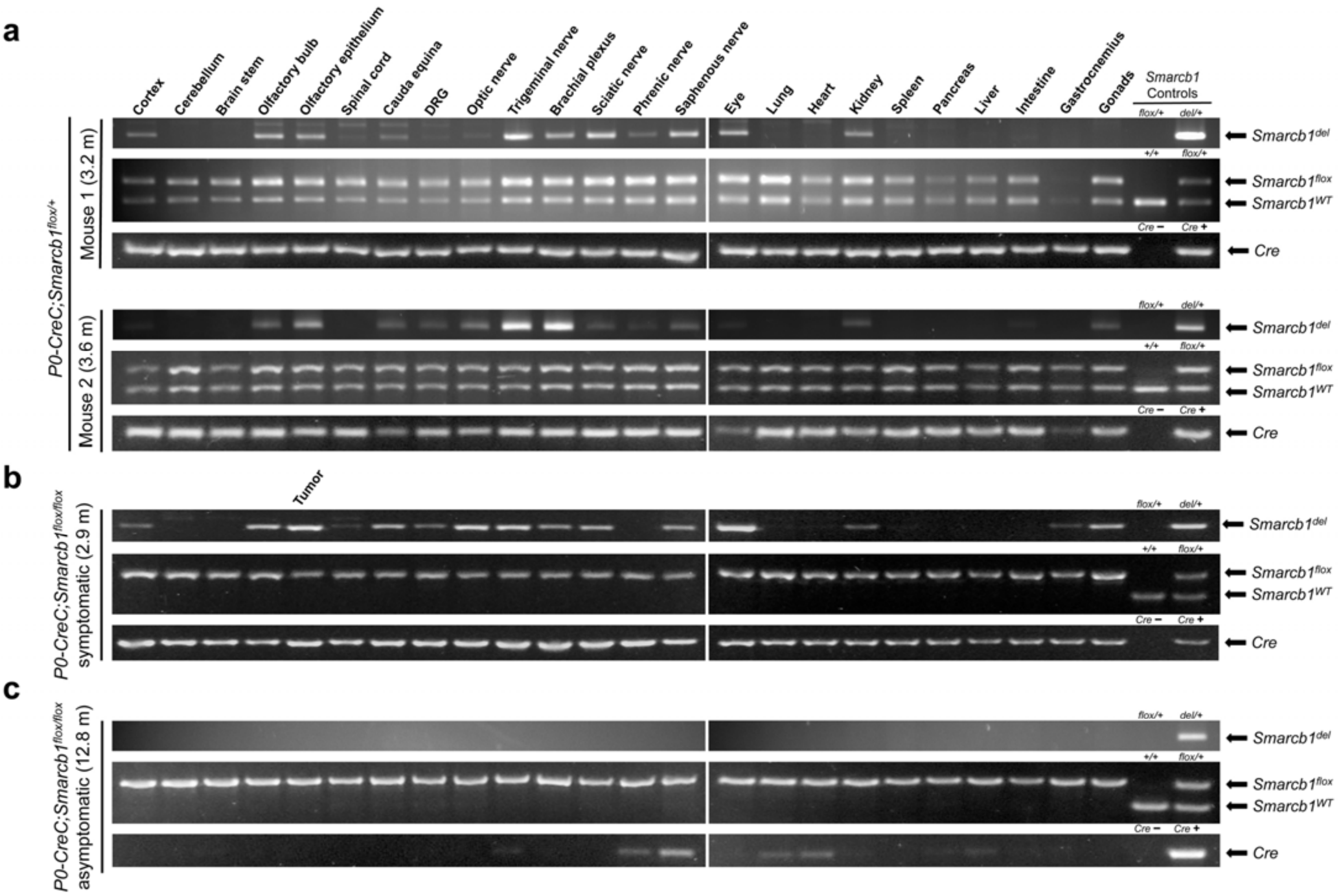

Supplementary Figure 2. Correlation of tumor location and Cre recombinase activity. (**a**) Detection of deleted *Smarcb1* allele in *P0-CreC;Smarcb1*$^{flox/+}$ mice correlated with tumor location in *P0-CreC;Smarcb1*$^{flox/flox}$ mice. Note that tissue-specific variability between the two different *P0-CreC;Smarcb1*$^{flox/+}$ mice explains interindividual variability of tumor location. (**b**,**c**) Detection of deleted, floxed and wild type (WT) *Smarcb1* alleles and *Cre* allele in various tissues of a young symptomatic *P0-CreC;Smarcb1*$^{flox/flox}$ mouse (**b**) and an asymptomatic *P0-CreC;Smarcb1*$^{flox/flox}$ mouse over 12 months of age (**c**). In (**b**), the olfactory epithelium tissue was replaced by the tumor tissue dissected from the nasal cavities.

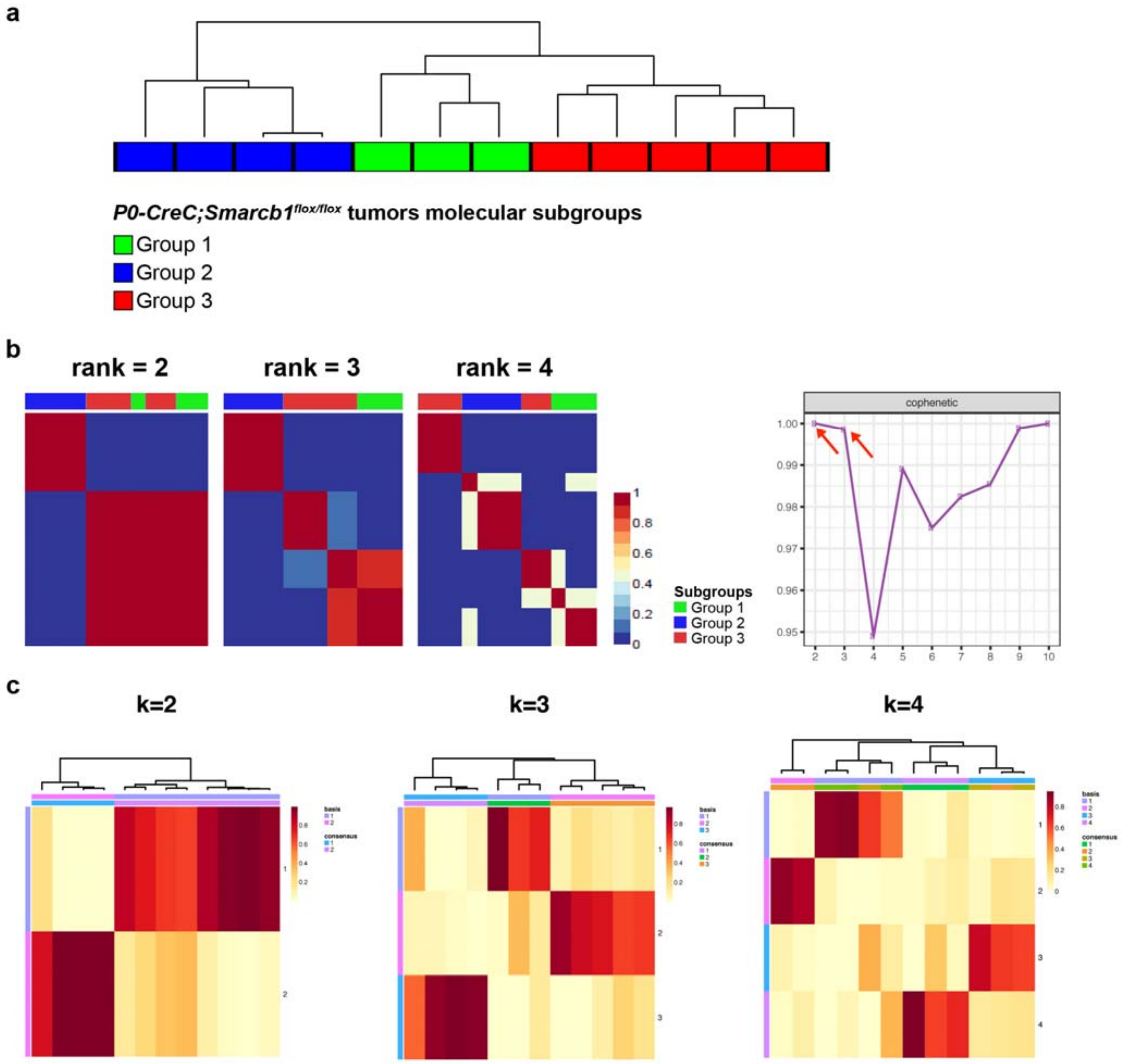

Supplementary Figure 3. Determination of the number of clusters for *P0-CreC;Smarcb1*$^{flox/flox}$ RTs. (**a**) Unsupervised hierarchical clustering based on the top 5000 most variable transcripts showed the clustering of 12 RTs from *P0-CreC;Smarcb1*$^{flox/flox}$ mice in three molecular subgroups. (**b**) Consensus matrices using the non-negative matrix factorization (NMF) method for ranks 2 to 4. Based on the maximum cophenetic correlation score, the optimal numbers of clusters is 2 or 3 (arrows). (**c**) Resulting heatmaps of the mixture coefficient matrices for k=2, k=3, and k=4.

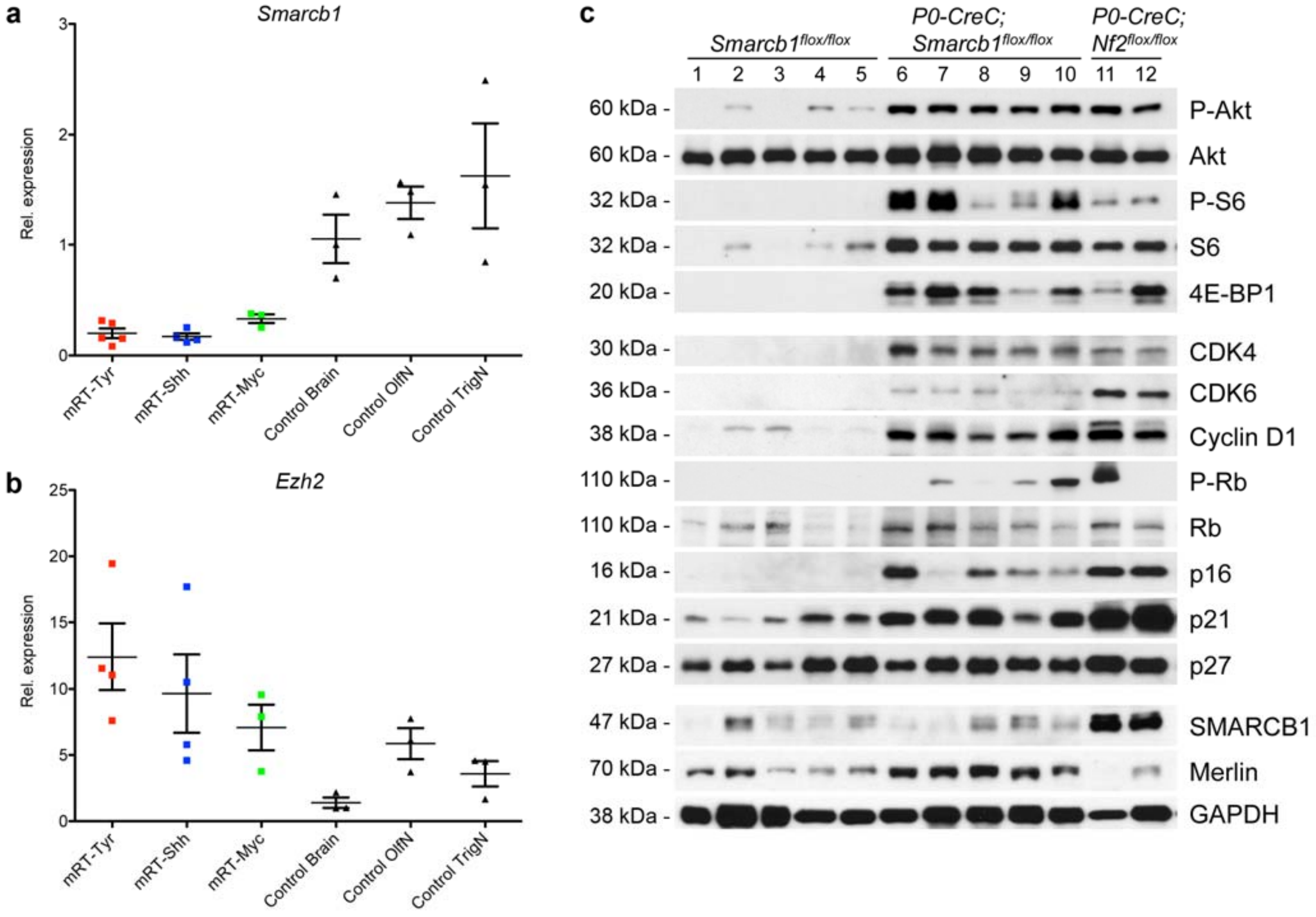

Supplementary Figure 4. Molecular analysis of RT from *P0-CreC;Smarcb1*$^{flox/flox}$ mice. (**a**,**b**) Relative expression level of *Smarcb1* (**a**) and *Ezh2* (**b**) in the three RT molecular subgroups and in control brain, olfactory (OlfN) and trigeminal (TrigN) nerves samples. Values represent mean ± s.e.m., each dot represent one tumor sample (**c**) Western blot showing protein level of tumors from *P0-CreC;Smarcb1*$^{flox/flox}$ mice located in the trigeminal (samples 6 and 7), olfactory (samples 8 and 9) or oculomotor nerves (sample 10) are compared to cortex (sample 1), olfactory bulbs (samples 2 and 3) and trigeminal nerves (samples 4 and 5) from *Smarcb1*$^{flox/flox}$ mice and schwannomas (samples 11 and 12) from *P0-CreC;Nf2*$^{flox/flox}$ mice. The same amount of protein (in µg) was loaded for each sample. The western blot shows activation of the Akt/mTOR and retinoblastoma signaling pathways in the tumors from *P0-CreC;Smarcb1*$^{flox/flox}$ mice. All uncropped scans of western blots are provided in Supplementary Fig. 7.

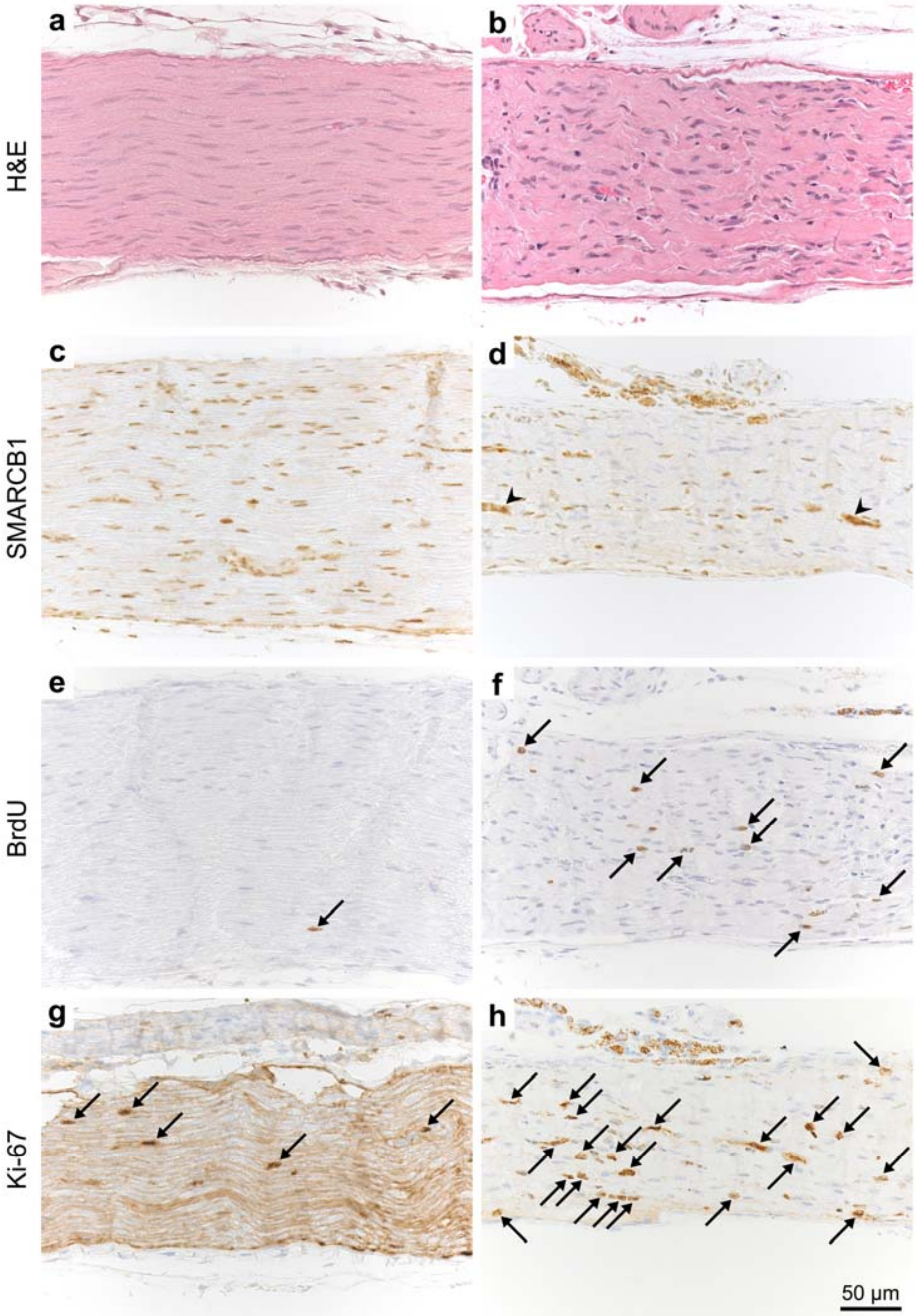

Supplementary Figure 5. *DHH-Cre;Smarcb1$^{flox/flox}$* mice reveal alteration of myelination and increased proliferation in sciatic nerves. (**a**,**b**) Hematoxylin and eosin staining shows disorganization of myelination in sciatic nerve from *DHH-Cre;Smarcb1$^{flox/flox}$* mice (**b**) compared to an age-matched *Smarcb1$^{flox/flox}$* control mouse (**a**). (**c**,**d**) SMARCB1 immunohistochemistry shows that most of the cells are SMARCB1 deficient in *DHH-Cre;Smarcb1$^{flox/flox}$* sciatic nerve (**d**), except endothelial cells (arrowheads). (**e**,**f**) BrdU staining shows increased number of proliferating cells (arrows) in sciatic nerve from *DHH-Cre;Smarcb1$^{flox/flox}$* mice (**f**, 7.9 ± 1.2%) compared to control (**e**, 0.8 ± 0.5%). (**g**,**h**) Ki-67 staining demonstrates an increased number of positive nuclei (arrows) in the sciatic nerve from *DHH-Cre;Smarcb1$^{flox/flox}$* mice (**h**, 15.4 ± 4.1%) compared to control (**g**, 2.1 ± 0.8%).

a

	mGFAP-Cre;Smarcb1$^{flox/flox}$ or *mGFAP-Cre;Smarcb1$^{del/flox}$*	*mGFAP-Cre;Smarcb1$^{flox/+}$*	*Smarcb1$^{flox/flox}$*	*Smarcb1$^{flox/+}$*
Alive at weaning	23.1%	24.1%	25.3%	27.5%
Expected	25%	25%	25%	25%

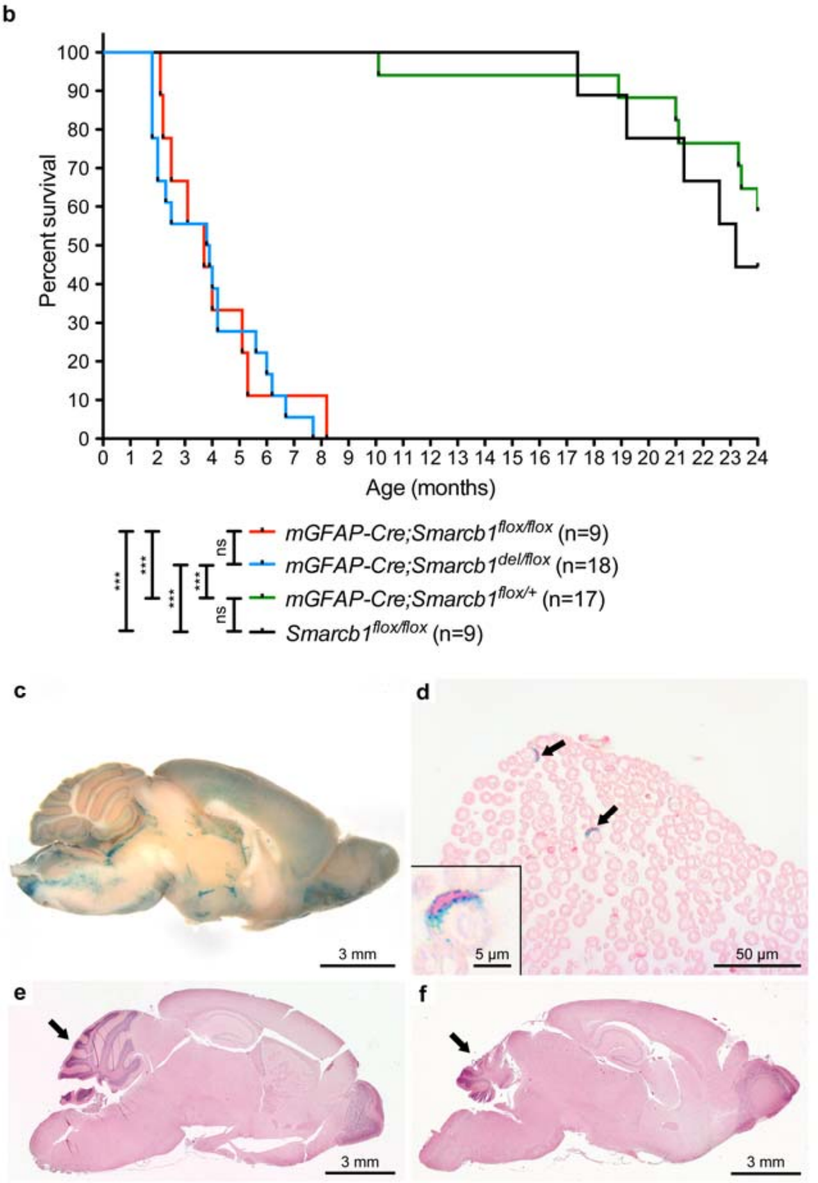

Supplementary Figure 6. *mGFAP-Cre;Smarcb1$^{flox/flox}$* mice develop severe motor deficits and ataxia phenotype. (**a**) Genotype distribution of mice at 3 weeks of age (n=229) from crossings of *mGFAP-Cre;Smarcb1$^{flox/+}$* and *Smarcb1$^{flox/flox}$* mice. *mGFAP-Cre;Smarcb1$^{flox/flox}$* and *mGFAP-Cre;Smarcb1$^{del/flox}$* mice were phenotypically indistinguishable and obtained at the expected Mendelian ratio (23.1% vs. 25%, non-statistically significant difference). (**b**) Kaplan-Meier curve representing the percent survival of *mGFAP-Cre;Smarcb1$^{flox/flox}$* (n=9, median survival = 3.7 months), *mGFAP-Cre;Smarcb1$^{del/flox}$* (n=18, median survival = 3.8 months), *mGFAP-Cre;Smarcb1$^{flox/+}$* (n=17, median survival = 23.2 months) and *Smarcb1$^{flox/flox}$* (n=9, median survival >24 months) mice versus age in months. (**c**) X-gal staining of a whole brain from a *mGFAP-Cre;AZCL* mouse shows *Cre* activation throughout the brain. (**d**) X-gal staining of a spinal nerve root section from a *mGFAP-Cre;AZCL* mouse demonstrating *Cre* activity in SCs (arrows). (**e**,**f**) Hematoxylin and eosin staining of brain sections shows the hypoplastic cerebellum (arrow) of a *mGFAP-Cre;Smarcb1$^{flox/flox}$* affected mouse (**f**) compared to a *Smarcb1$^{flox/flox}$* control mouse (**e**). ns (non significant), *** ($p<0.001$); log-rank (Mantel-Cox) test.

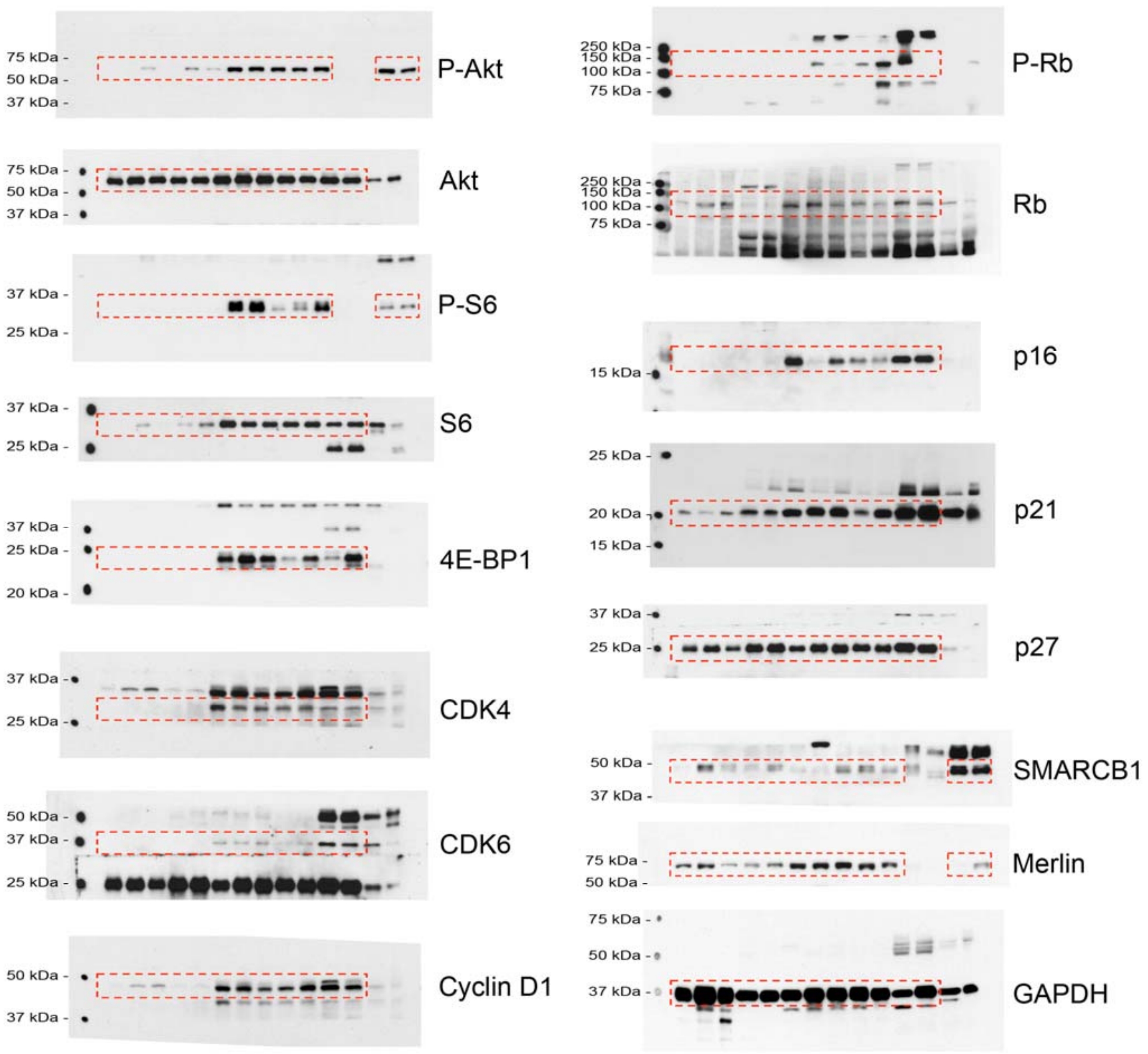

Supplementary Figure 7: Uncropped scans of western blots included in Supplementary Fig. 4. The cropped regions are indicated by red boxes.

	P0-CreC;Smarcb1$^{flox/flox}$ (Rhabdoid tumors)							*P0-CreC;Nf2*$^{flox/flox}$ (Schwannomas)		
	FABP7	GFAP	S100	Vimentin	SMA	NF	Cytokeratin	FABP7	GFAP	S100
Strong/majority of positive cells	7/9	0/9	0/9	6/9	2/9	0/9	0/9	3/7	1/7	6/7
Moderate/foci of positive cells	2/9	1/9	0/9	3/9	4/9	0/9	0/9	4/7	4/7	1/7
Weak/few positive cells	0/9	0/9	3/9	0/9	3/9	0/9	0/9	0/7	2/7	0/7
No staining	0/9	8/9	6/9	0/9	0/9	9/9	9/9	0/7	0/7	0/7

Supplementary Table 1. Immunohistochemical analysis of RTs from *P0-CreC;Smarcb1*$^{flox/flox}$ mice (n=9) and schwannomas from *P0-CreC;Nf2*$^{flox/flox}$ mice (n=7).
